# Deep Learning for Fully Automated Radiographic Measurements of the Pelvis and Hip

**DOI:** 10.3390/diagnostics13030497

**Published:** 2023-01-29

**Authors:** Christoph Stotter, Thomas Klestil, Christoph Röder, Philippe Reuter, Kenneth Chen, Robert Emprechtinger, Allan Hummer, Christoph Salzlechner, Matthew DiFranco, Stefan Nehrer

**Affiliations:** 1 Department for Orthopedics and Traumatology, Landesklinikum Baden-Mödling, 2340 Mödling, Austria; 2 Department for Health Sciences, Medicine and Research, University for Continuing Education Krems, 3500 Krems, Austria; 3 ImageBiopsy Lab, 1140 Vienna, Austria

**Keywords:** femoroacetabular impingement, hip dysplasia, X-ray, radiographs, artificial intelligence, machine learning, neuronal networks

## Abstract

The morphometry of the hip and pelvis can be evaluated in native radiographs. Artificial-intelligence-assisted analyses provide objective, accurate, and reproducible results. This study investigates the performance of an artificial intelligence (AI)-based software using deep learning algorithms to measure radiological parameters that identify femoroacetabular impingement and hip dysplasia. Sixty-two radiographs (124 hips) were manually evaluated by three observers and fully automated analyses were performed by an AI-driven software (HIPPO™, ImageBiopsy Lab, Vienna, Austria). We compared the performance of the three human readers with the HIPPO™ using a Bayesian mixed model. For this purpose, we used the absolute deviation from the median ratings of all readers and HIPPO™. Our results indicate a high probability that the AI-driven software ranks better than at least one manual reader for the majority of outcome measures. Hence, fully automated analyses could provide reproducible results and facilitate identifying radiographic signs of hip disorders.

## 1. Introduction

Hip dysplasia and femoroacetabular impingement (FAI) are risk factors for the development of osteoarthritis of the hip [1,2]. These disorders often occur concomitantly, and early diagnosis is crucial so that treatment may be initiated before permanent damage appears [3,4].

In 2003, Ganz et al. provided a comprehensive overview on the concept of femoroacetabular impingement [5]. Our understanding of the pathogenesis has grown significantly since then. FAI is defined as a dynamic conflict of the proximal femur and the acetabulum, caused by early engagement during motion, mainly flexion and internal rotation of the hip [5]. Intra-articular impingement is subdivided in cam- and pincer-type FAI, although combinations frequently occur [1]. Additionally, the femoral torsion contributes to the development of FAI. Cam-type FAI is caused by a deformity at the antero-superior femoral head–neck junction with an aspherical contour that produces compression and shearing forces at the labrum and articular cartilage of the acetabulum [6]. When it remains untreated, this can lead to chondro-labral separation, degeneration of the labrum, and the delamination of the articular cartilage. Pincer-type FAI is characterized by an excessive acetabular coverage of the femoral head that results in a compression of the labrum between the acetabulum and the femoral neck. Pincer deformity can be caused by deep acetabula (i.e., protrusion) or a prominent anterior wall (i.e., retroversion). Regardless of the type of deformity, untreated FAI causes damage to the hip and osteoarthritis may develop. Symptomatic FAI in adolescents should primarily be treated non-operatively. However, hip arthroscopy shows significant improvement of clinical symptoms and high return-to-sport rates [3].

Hip dysplasia is defined by a reduced acetabular coverage of the femoral head, frequently with a decreased lateral center-edge angle and an increased acetabular index. This leads to a decreased contact area of the load-bearing articular cartilage and increased contact pressures [7]. The prevalence of hip dysplasia in an asymptomatic population is reported to be between 3.6% and 12.8%, depending on the radiographic applied [8,9]. Known risk factors are female sex, breech presentation, and family history [10]. For joint preservation in young patients, a surgical correction of hip dysplasia osteotomies of the acetabulum is performed with additional hip arthroscopy to address intraarticular pathologies.

The diagnoses of FAI and hip dysplasia are primarily made clinically by detecting a decreased range of motion and pain with flexion and internal rotation. However, imaging plays a crucial role in the quantitative deformity analysis and preoperative assessment. Plain radiographs still represent the gold standard, followed by more advanced imaging techniques such as magnetic resonance imaging (MRI) or magnetic resonance (MR)-arthrography [11]. Standard imaging includes conventional radiographs (antero-posterior (AP) pelvis, Lauenstein view, or Faux profile) to evaluate the geometry and morphometry of the hip joint, including acetabular coverage and the asphericity of the femoral head.

The manual measurement of these parameters is a tedious and time-consuming task which demonstrates high inter- and intra-observer differences [12]. Automated, artificial-intelligence-assisted analyses could provide objective, highly accurate, and reproducible results when compared to manual readers [13,14]. However, AI analyses depend on the training data used and potential bias could be introduced. When investigated for reliability and agreement, measurements that could be performed directly showed better results than those that needed estimation, such as the acetabular index or caput-collum-diaphyseal (CCD) angle [15]. Furthermore, it has been shown that the agreement rate of orthopedic surgeons and radiologists is good within their specialty, but simultaneously reflects low reliability between different specialties [16].

The aim of the present work was to investigate the performance of an AI-driven software in analyzing the most common radiographic parameters for hip and pelvic morphology compared to manual measurements.

## 2. Materials and Methods

This retrospective study was approved by the Lower Austria ethics committee (GS4-EK-3/173-2020). Native, weight-bearing AP radiographs of the pelvis were collected for this study. These were consecutively acquired between November 2019 and January 2020 at the Landesklinikum Baden-Mödling. Individual informed consent was waived by the ethics committee due to the retrospective study design and the pseudonymization of the data. Inclusion criteria were defined as male and female adults aged between 18 and 60 years and radiographs which complied with the quality standards. Image quality was assessed before readers started the annotation process. The assessment included checks for incorrect image cropping, clear visibility of bone contours, and excessive tilt and rotation, as well as a tilted sensor. Exclusion criteria included severe deformities, detectable surgical implants, and post-traumatic cases. All radiographs were acquired with the same device (DigitalDiagnost, Philips).

### 2.1. Manual Measurement

**Figure 1:**
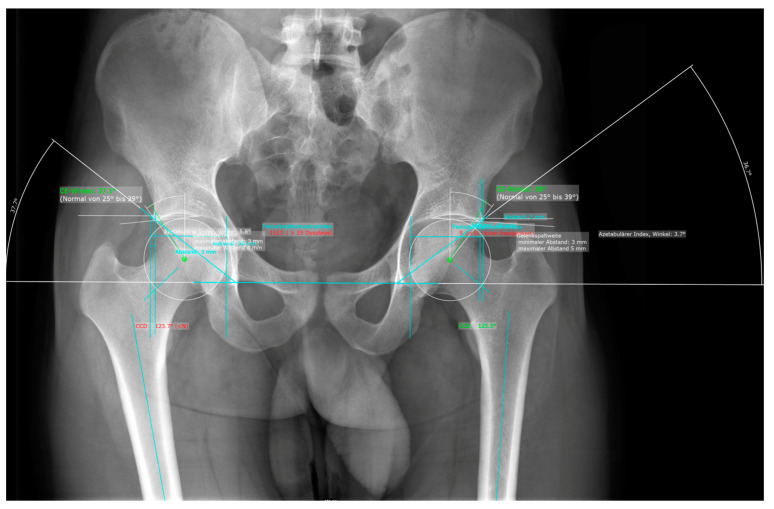
Representative image of the manual measurements for an AP radiograph of the pelvis using mediCAD^®^.

Manual measurements were carried out independently by three investigators (C.S., C.R., P.R.). All investigators were orthopedic surgeons with a minimum of five years’ experience in musculoskeletal imaging. The annotations were obtained using mediCAD® (FAI module v6.0, mediCAD Hectec GmbH, Altdorf/Landshut, Germany), according to the user’s manual workflow (Figure 1). Each reader was blinded to the AI results, worked independently, and annotated each image in the same order.

### 2.2. Automated Measurements Using AI Software

**Figure 2:**
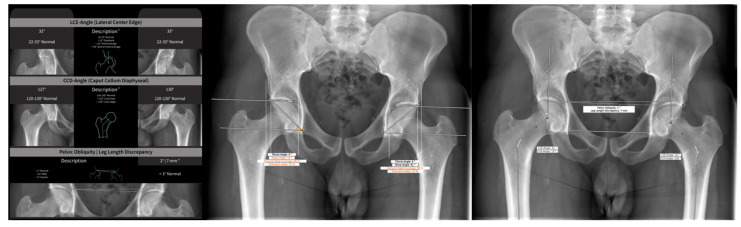
AI software (HIPPO™) report of an AP radiograph of the pelvis providing fully automated measurements.

Automated analyses were accomplished by using a commercially available, AI-based software (HIPPO™, CE version, HIP Positioning Assistant, ImageBiopsy Lab, Vienna, Austria). HIPPO™ was developed using deep learning algorithms and trained on over 4000 individual radiographs of the pelvis and hip. Radiographs for the training data were acquired from a total of three sources: the Osteoarthritis Initiative study, the Cohort Hip and Cohort Knee study, and from an orthopedic hospital in Austria. The readers of the present study were not involved in generating the training dataset. HIPPO™ automatically detects and localizes anatomically relevant landmarks on the hip and pelvis. The AI follows the established radiological workflow: measurement of anatomical distances and angles, detection of disease morphologies, and provision of standardized reporting (Figure 2). HIPPO™ performs a consensus assessment for each radiograph. Every detection step is performed by three AI models, which then vote for the appropriate result. IB Lab HIPPO™ is comprised of multiple convolutional deep neural networks (CNNs) which operate on either all or part of the input images and perform segmentation, landmarking, and detection tasks. A detailed description of the calculation logic and the CNNs is provided in Supplement File S1.

### 2.3. Measurements

**Figure 3:**
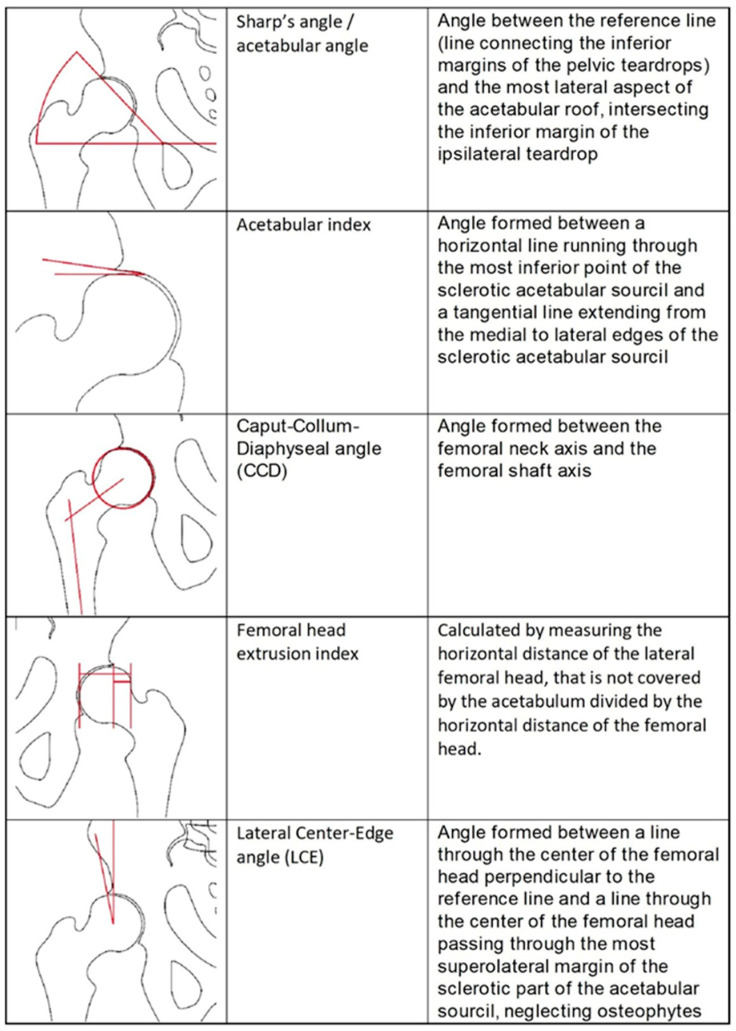
Radiographic measurement for the evaluation of the hip morphology used in this study.

To evaluate the geometry and morphometry of the hip joint and pelvis, the following measurements were performed: CCD angle, lateral center-edge (LCE) angle, acetabular index (Tönnis angle and sourcil angle), femoral head extrusion index, and Sharp angle (Figure 3).

### 2.4. Statistical Analysis

We used a Bayesian approach in our analysis, which has several advantages over conventional frequentist methods. These advantages include an ease of interpretation and the avoidance of issues related to null hypothesis significance testing. In our case, the Bayesian approach allowed us to compare the performance of the individual human readers with the AI and account for the fact that there is no real ground truth available. For this purpose, we used the surface under the cumulative ranking (SUCRA) metric. We ranked the readers and the AI according to the absolute deviation from the median of ratings from all readers and the AI. To measure performance, we used the SUCRA metric. We ranked the readers and the AI based on the absolute deviation from the median of ratings from all readers and the AI. The lowest possible rank of four readers was four, and a probability of 50% of a rank of two in the plot indicates that the probability for a specific reader to rank at least place two was 50%. All analyses were conducted in the R environment (version 4.2.1) using the tidyverse package for data wrangling and plot creation. The calculations were performed using the Markov chain Monte Carlo via the brms package. We used restrictive priors for our analyses, preventing negative values for the absolute deviation. We calculated an interaction model (reader by outcome) with suppressed intercept. The model settings in specific were:

$$Absolute\;Deviation_{i}\;\sim \;Normal\left(\mu_{i},\;\sigma \right)\;\mu_{i}=\gamma_{reader\left[i\right]}\times \beta_{outcome\left[j\right]}\;\gamma_{i},\;\beta_{j}\;\sim \;Lognormal\left(0,\;3\right),\mathrm{for}\;i\;=\;1,\;\ldots ,\;4\;\mathrm{and}\;j\;=\;1,\;\ldots ,\;6\;\sigma_{\gamma },\;\sigma_{\beta }\sim \;HalfCauchy\left(0,\;4\right)$$

## 3. Results

**Figure 4:**
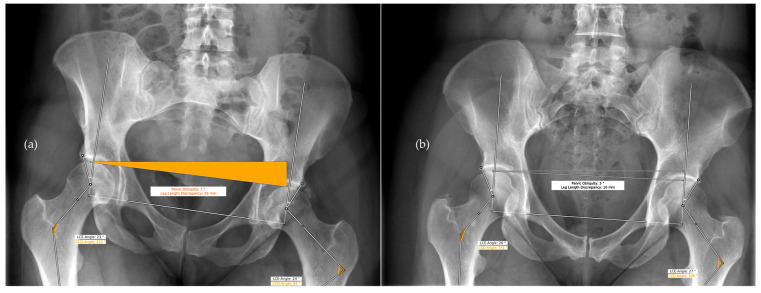
HIPPO™ erroneous reports of two AP radiographs of the pelvis that were identified as outliers. (**a**) Pelvic obliquity resulted in a cropped proximal femur and the anatomical femur axis could not be identified correctly. (**b**) The proximal femur is barely visible and the anatomical femur axis could not be identified correctly.

A total of 62 radiographs (124 hips) were included in this study (age: 36.9 ± 11.6 years; 34 female, 28 male). Two outliers were identified, which were caused by an erroneous analysis by the AI software (Figure 4). The following plots exclude these outliers. An analysis including the outliers is provided in Supplement File S2.

**Figure 5:**
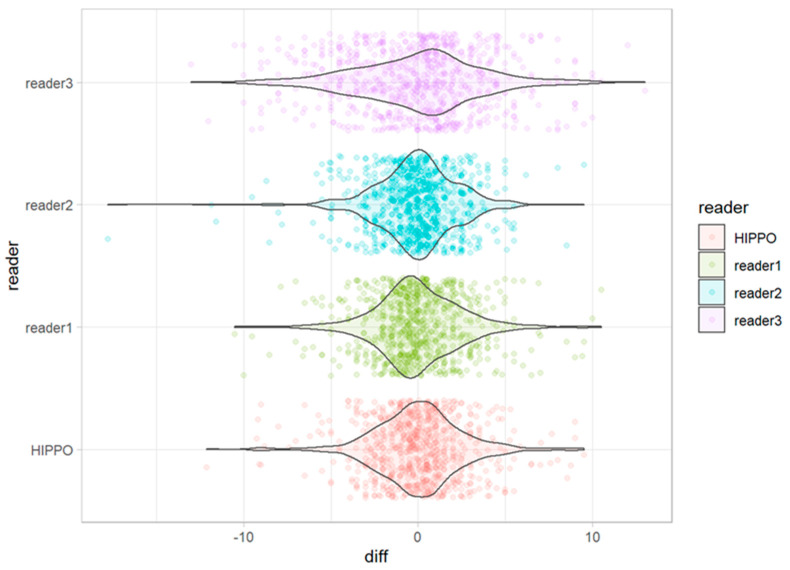
Deviation from the median for each individual observation for all readers and HIPPO™.

**Figure 6:**
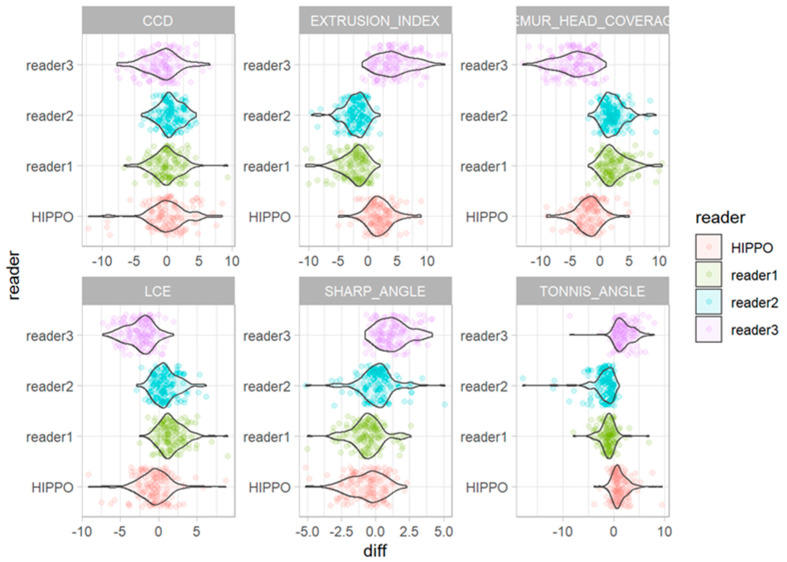
Deviations from the median for each individual observation for all measurements for readers one to three and HIPPO™.

**Figure 7:**
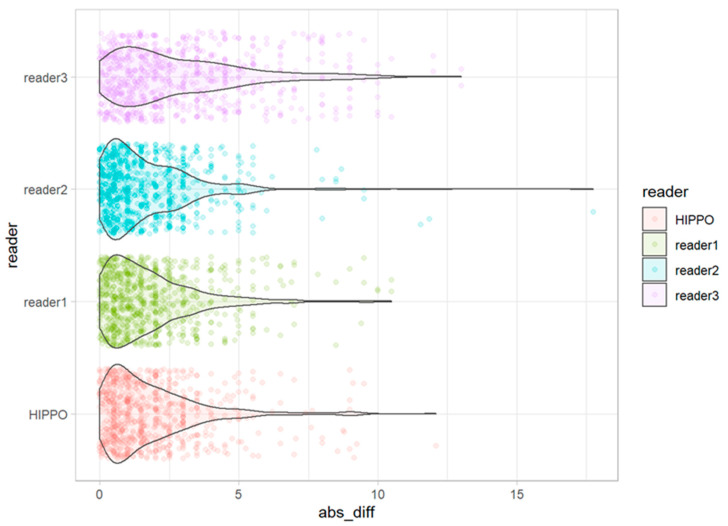
Absolute deviation from the median for each individual observation for all readers and HIPPO™.

**Figure 8:**
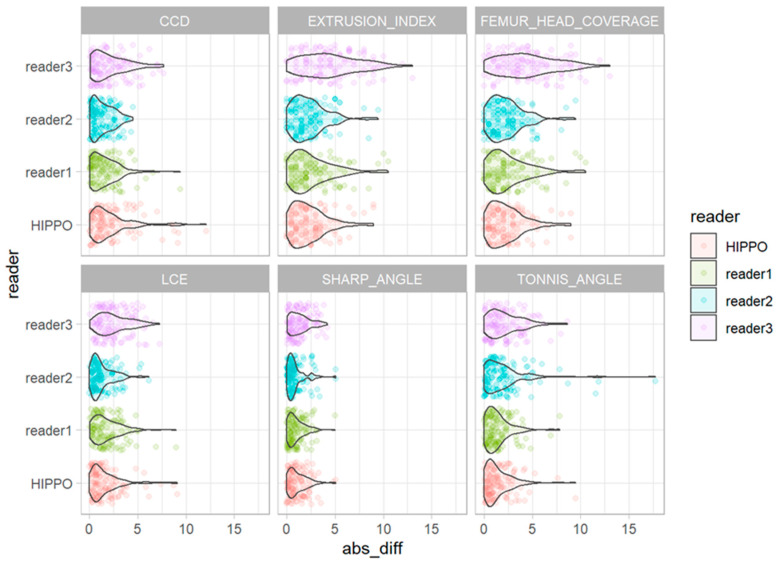
Absolute deviation from the median for all measurements for all readers and HIPPO™.

**Figure 9:**
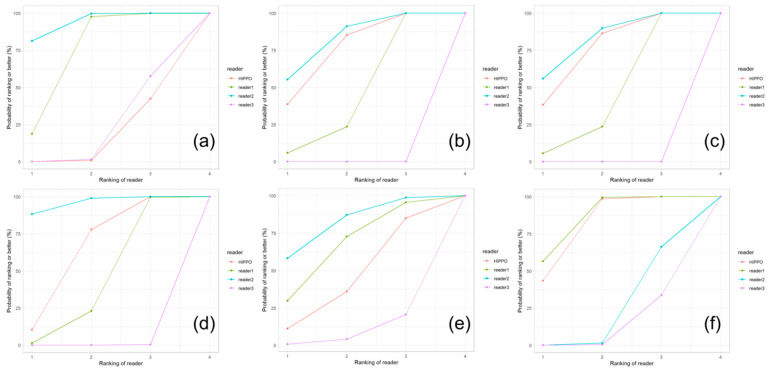
SUCRA (surface under the cumulative ranking) plots for (**a**) CCD, (**b**) extrusion index, (**c**) femur head coverage, (**d**) LCE, (**e**) Sharp angle, and (**f**) Tönnis angle. The plots indicate the probabilities that a reader ranks better (i.e., less absolute deviation from the median) than a certain rank.

The deviations from the median for all observations are displayed in Figure 5. The deviations from the median for each outcome measurement and all readers are displayed in Figure 6. The corresponding absolute deviations are displayed in Figure 7 and Figure 8. The SUCRA plots show the probabilities that an individual reader ranks better (meaning less absolute deviation from the median) than a certain rank (Figure 9). Except for the CCD, the AI software showed high probabilities to outperform at least one manual reader. For the extrusion index, the femur head coverage, the LCE, and the acetabular index, the probability for the software to rank at least place three was nearly 100%; for the Sharp angle, it was over 80%. The detailed results for the SUCRA plots are provided in Supplement File S2. Our models indicate a good fit (Rhat = 1.00; Bulk_ESS ≥ 7323; and Tail_ESS ≥ 2374).

## 4. Discussion

The main finding of this study was that the AI-based software produced reliable results for common radiographic parameters when determining the morphology of the hip and pelvis. In addition, when compared with the manual measurements, the AI-results showed a high probability to perform better than at least one manual reader for all measurements except CCD.

A profound radiographic evaluation is mandatory in patients with FAI and hip dysplasia. Both pathologies are associated with early-onset osteoarthritis of the hip, and early detection allows for joint-preserving procedures such as periacetabular osteotomies or hip arthroscopy [2,3]. Analyses are usually performed manually by a radiologist or an orthopedic surgeon. The standard manual workflow for radiographic analysis of the hip and pelvic morphology using a commercially available medical software involves: identifying the hip joint center, defining a reference line for the pelvic orientation, and measuring the CCD angle, LCE angle, the acetabular index, the femoral head extrusion index, and the Sharp angle.

The AI-driven software used in this study includes multiple convolutional deep neural networks that perform segmentation, landmarking, and detection. Anatomical landmarks are detected fully automated and every detection step is performed by three AI models simultaneously that then vote for a result. The software was developed using deep learning algorithms. Deep learning goes beyond machine learning as it uses neural networks [17]. In deep learning, large amounts of data can be processed and analyzed and, by using neural networks, information that already exists can be interpreted and further processed. Acquired information can be merged with new data to be used for future applications. An increasing number of publications investigate AI-driven software for various diagnostic applications and outcome prediction across all medical disciplines. In the field of orthopedics, these applications include fracture detection, classification of osteoarthritis and bone age, and automated measurements of the lower extremities [18]. AI applications for hip radiographs include the assessment of hip arthroplasties, fracture detection, and the automated detection of anatomical landmarks [19,20,21,22,23].

In a study investigating the classification of hip fractures, a machine learning method achieved an overall accuracy of 92% and was able to classify hip fractures with a 19% greater accuracy than humans [19]. However, the applied software was a prototype and is not ready for clinical use.

Recently, two publications investigated radiographic signs of hip dysplasia on ap radiographs of the pelvis [13,14]. Archer et al. used the same AI-based software in an external validation study to assess patients with proven adult hip dysplasia [14]. Three manual reader’s measurements were compared to AI measurements for the measurements provided by HIPPO™. The authors choose conventional frequentist methods for statistical analyses. The inter-reader analysis demonstrated fair to excellent agreement. However, for several analyses, including of the Tönnis angle and CCD, wide confidence intervals were observed. When applying an AI software for radiographic measurements, the results are often compared with a “ground truth” that is defined by manual readers. This approach has various disadvantages, including the inaccuracy caused by a high interrater variability that deteriorates the ground truth. In order to acquire a ground truth, the number of manual readers would have to be high and should include only specialists that ideally reach agreement for every observation and every measurement. Therefore, to account for these shortcomings, we used a Bayesian approach to compare the performance of the individual human readers with the AI-driven software. Compared with conventional frequentist methods, this approach has several advantages, including facilitation of interpretation and the avoidance of issues related to null-hypothesis significance testing. As the authors used the same commercially available and CE-certified software, the analyses were carried out in a standardized fashion. However, this study investigated proven cases of hip dysplasia and patients without normal hip anatomy without pathological findings were not included.

In a similar approach, Jensen et al. tested a newly developed deep learning algorithm for the radiographic measurement of the hip (RBhip™, Radiobotics). The agreement between the algorithm and five human readers for measuring the LCE angle and the acetabular index was investigated. In accordance with the available literature, the manual measurements were susceptible to high inter-reader differences and the level of agreement between the algorithm and manual readers was poor [13].

Jang et al. developed and evaluated an automated measurement model for ap pelvic radiographs [20]. After training, the CNN model was able to define anatomical landmarks without manual labeling, and these landmarks were used to calculate the femoral head extrusion index, Sharp angle, Tönnis angle, and CE angle of Wiberg using automatic algorithms. The percentage of correct key points with a 3mm threshold ranged from 87% to 100%, and the intraclass correlation between the model and the reference standard was 0.83 to 0.93.

Table 1 provides an overview of recent studies using deep learning approaches for hip radiographs.

However, there is still a paucity of prospective studies and randomized trials for deep learning applications in musculoskeletal imaging in the present literature [24]. The majority of existing studies are not prospective, contain a high risk of bias, and do not use reporting standards. Furthermore, manual comparison groups are often small and studies develop and test deep learning algorithms without open-source access.

Amongst other factors, AI was introduced in orthopedics to reduce the human failure rate and increase reproducibility. In this study, the manual analysis of a bilateral hip image took approximately six minutes per radiograph. In contrast, the automated measurements and resulting standardized report by the AI-driven software was completed within under 30 s. These time-saving effects support previous reports [14]. Furthermore, manual readers show elevated rates of errors with fatigue [25,26]. Independent of experience and fatigue, AI reduces the impact of interrater variability in radiographic morphology assessment of the hip.

For all observations, mean deviations from the median showed an even distribution for all readers and HIPPO™. However, when disaggregated for the different measurements, differences between the readers become apparent. For the CCD, the manual readers and HIPPO™ showed a similar distribution of observations with an even spread around the mean. In contrast, for measurements that included the labeling of the lateral acetabular edge, we observed differences between the individual manual readers, indicating a methodical deviation. For these measurements, the AI-based software showed values in between the manual readers. The software was trained on over 4000 individual radiographs acquired from large international cohorts and automatically detects and localizes anatomically relevant landmarks. Thereby, the AI performs a consensus assessment for three AI models for each radiograph and is not prone to subjective assessments and ratings. Our analyses indicate that individual readers might be susceptible to systematic disagreement that result in either positive or negative deviations from the median. The AI software showed no deviation in any direction greater than a manual reader. The SUCRA plots indicate a non-inferiority for the AI-driven software.

In our study, the AI software showed erroneous measurements for two hips. In both cases, the anatomical femur axis could not be localized correctly due to pelvic obliquity and excessive cropping of the proximal femur. Hence, the CCD showed incorrect values, while the measurements based on the femoral head and acetabulum were not affected. In this context, it needs to be emphasized that, in the current state of development, all automated measurements performed by an AI software need to be checked and confirmed by the user.

Currently, properly acquired radiographs are essential for the precise analysis of radiological signs of FAI and dysplasia, as there is a high variability when comparing pelvic-focused views and radiographs acquired in a supine position. In the future, with enough training and validation data, AI-driven software might be able to compensate for poor image quality. Although more advanced imaging techniques, such as MRI or MR-arthrography, are in use for the diagnosis of hip disorders and show higher sensitivities, plain radiography with hip projections remains the basic diagnostic imaging tool [11]. AI applications are capable of processing large numbers of images very quickly and can be used for standardized and reproducible analysis.

Our results demonstrate that the most common radiographic parameters for FAI and hip dysplasia can be determined in a fully automated method with an accuracy comparable to manual readers. 

This study has some limitations. First, the parameters that were evaluated in this study do not cover the complete radiological analysis for FAI and hip dysplasia. For instance, the crossover sign to identify acetabular retroversion was not assessed and measurements were performed on AP radiographs. For Cam-type FAI, a Dunn view projection is typically also used for detecting femoral head–neck asphericity with increased sensitivity. The quality assessment for the inclusion of radiographs for this study was performed manually and did not include objective ratings for pelvic obliquity or malrotation, resulting in a potential risk for selection bias. Furthermore, the manual measurements in this study were performed only by orthopedic surgeons, introducing a potential bias in the analyses. Hence, the reliability between different specialties could not be investigated.

## 5. Conclusions

An AI-driven software can provide fully automated measurements of native, weight-bearing AP radiographs of the pelvis with great accuracy and reproducibility. Using deep learning algorithms can facilitate the identification of radiographic signs of femoroacetabular impingement and hip dysplasia. However, diagnoses need to be confirmed by medical professionals.

## Figures and Tables

**Table 1:** Selection of studies on artificial intelligence and measurements of the hip. ap = anterior-posterior CE = center-edge angle of Wiberg, FHEI = femoral head extrusion index, PCK = percentage of correct key points, ICC = intraclass correlation index, r = Pearson’s coefficient, RMSE = root mean square error, MAE = mean absolute error, CCD = caput-collum-diaphyseal angle, THA = total hip arthroplasty, DSC = Dice similarity coefficient, DL = deep learning, AIA = acetabular index angle, LOA = limits of agreement.

Reference	Purpose	Method	Results and Performance
Jang et al., 2022 [20]	Automated determination of hip joint center	U-Net used for identification of bony landmarks and pelvic height ratio. A total of 6344 ap hip radiographs used for training, and used 1252 for testing. Compared to manual segmentation.	Prediction within 5 mm of error: 80% with nonspecific, 83% sex-specific. And 91% with patient-specific pelvic height ratio.
Yang et al., 2020 [22]	Feasibility Study for automated measurement of the hip joint (determination of CE, Tönnis angle, Sharp angle, FHEI)	Identification of bony landmarks. A total of 1060 ap hip radiographs used for training, and 200 used for testing. Compared to three radiologists.	PCK: 87–100%, ICC: 0.8–0.93, r: 0.83–0.93, RMSE: 0.02–3.27, MAE: 0.02–1.79.
Archer et al., 2022 [14]	Detection of Hip dysplasia through lateral CE, CCD, pelvic obliquity, Tönnis angle, Sharp angle, femoral head coverage using HIPPO™	HIPPO™ used for Identification of bony landmarks. 256 ap hip radiographs for testing. Compared to three medical students who underwent instructions form one senior radiologist.	ICC for lateral CE: 0.71–0.86, for CCD: 0.62–0.79, for pelvic obliquity: 0.83–0.98, for Tönnis angle: 0.82–0.90, for Sharp angle: 0.74–0.86, for femoral head coverage: 0.5–0.73.
Rouzrokh et al., 2021 [21]	Automated measurement of acetabular component and version after THA	2 U-Net models for Segmentation of bilateral ischial tuberosity on 600 ap hip radiographs and acetabular component on 600 ap and cross-table lateral hip radiographs. Training, validation and testing split in 8:1:1 ratio. Compared to two orthopedic surgeons.	For ap and cross-table lateral radiograph models, respectively: egmentation: mean DSC 0.878 and 0.903, Acetabular component angles: mean absolute difference 1.35° and 1.39°.
Rouzrokh et al., 2022 [23]	Creating THA radiography registry using deep learning	Four DL algorithms used for determination of radiograph appearance on 846,988 hip and pelvis radiographs. Compared to human annotators on random test sample of 5000 radiographs.	209,331 radiographs were excluded as misclassified. Accuracy: 99.9%, precision: 99.6%, recall: 99.5%, F1-score: 99.6%. Registry automatically annotated in <8 h
Jensen et al., 2022 [13]	Detection of hip dysplasia through lateral CE and AIA	RBHip™ trained on 2900 pelvic radiographs, tested on 71 pelvic radiographs. Comparison to ground truth: 5 clinicians.	Lateral CE: Bland–Altman LoA ranging from 0.37 to 9.56 and 3.56 to 10.1 for right and left hip, respectively. AIA: Bland-Altman LoA ranging from −0.58 to 2.06 and −1.09 to 1.28 for right and left hip, respectively.

## Data Availability

Not applicable.

## Supplementary Materials

The following supporting information can be downloaded at: https://www.mdpi.com/article/10.3390/diagnostics13030497/s1.

## References

[1] Vuillemin N., Steppacher S.D., Meier M.K., Büchler L. (2022). Therapieentscheidung bei Kombinationspathologien Dysplasie—FAI—Fehlrotation. Orthopädie.

[2] Melugin H.P., Hale R.F., Zhou J., LaPrade M., Bernard C., Leland D., Levy B.A., Krych A.J. (2020). Risk Factors for Long-term Hip Osteoarthritis in Patients with Femoroacetabular Impingement Without Surgical Intervention. Am. J. Sports Med..

[3] Chiari C., Lutschounig M.C., Nöbauer-Huhmann I., Windhager R. (2022). Femoroacetabular impingement syndrome in adolescents. Orthopade.

[4] Pascual-Garrido C., Li D.J., Grammatopoulos G., Yanik E.L., Clohisy J.C., ANCHOR Group (2019). The Pattern of Acetabular Cartilage Wear Is Hip Morphology-dependent and Patient Demographic-dependent. Clin. Orthop. Relat. Res..

[5] Ganz R., Parvizi J., Beck M., Leunig M., Nötzli H., Siebenrock K.A. (2003). Femoroacetabular impingement: A cause for osteoarthritis of the hip. Clin. Orthop. Relat. Res..

[6] Bech N.H., Haverkamp D. (2018). Impingement around the hip: Beyond cam and pincer. EFORT Open Rev..

[7] Gala L., Clohisy J.C., Beaulé P.E. (2016). Hip Dysplasia in the Young Adult. J. Bone Jt. Surg. Am..

[8] Gosvig K.K., Jacobsen S., Sonne-Holm S., Palm H., Troelsen A. (2010). Prevalence of malformations of the hip joint and their relationship to sex, groin pain, and risk of osteoarthritis: A population-based survey. J. Bone Jt. Surg. Am..

[9] Jacobsen S., Sonne-Holm S. (2005). Hip dysplasia: A significant risk factor for the development of hip osteoarthritis. A cross-sectional survey. Rheumatology.

[10] Bache C.E., Clegg J., Herron M. (2002). Risk factors for developmental dysplasia of the hip: Ultrasonographic findings in the neonatal period. J. Pediatr. Orthop..

[11] Schmaranzer F., Kheterpal A.B., Bredella M.A. (2021). Best Practices: Hip Femoroacetabular Impingement. Am. J. Roentgenol..

[12] Cadet E.R., Babatunde O.M., Gorroochurn P., Chan A.K., Stancato-Pasik A., Brown M., Johnson S., Kaiser P.B., Gardner T.R., Ayeni O.R. (2016). Inter- and intra-observer agreement of femoroacetabular impingement (FAI) parameters comparing plain radiographs and advanced, 3D computed tomographic (CT)-generated hip models in a surgical patient cohort. Knee Surg. Sports Traumatol. Arthrosc..

[13] Jensen J., Graumann O., Overgaard S., Gerke O., Lundemann M., Haubro M.H., Varnum C., Bak L., Rasmussen J., Olsen L.B. (2022). A Deep Learning Algorithm for Radiographic Measurements of the Hip in Adults-A Reliability and Agreement Study. Diagnostics.

[14] Archer H., Reine S., Alshaikhsalama A., Wells J., Kohli A., Vazquez L., Hummer A., DiFranco M.D., Ljuhar R., Xi Y. (2022). Artificial intelligence-generated hip radiological measurements are fast and adequate for reliable assessment of hip dysplasia: An external validation study. Bone Jt. Open.

[15] Mast N.H., Impellizzeri F., Keller S., Leunig M. (2011). Reliability and agreement of measures used in radiographic evaluation of the adult hip. Clin. Orthop. Relat. Res..

[16] Ayeni O.R., Chan K., Whelan D.B., Gandhi R., Williams D., Harish S., Choudur H., Chiavaras M.M., Karlsson J., Bhandari M. (2014). Diagnosing Femoroacetabular Impingement from Plain Radiographs: Do Radiologists and Orthopaedic Surgeons Differ?. Orthop. J. Sports Med..

[17] Sidey-Gibbons J.A.M., Sidey-Gibbons C.J. (2019). Machine learning in medicine: A practical introduction. BMC Med. Res. Methodol..

[18] Chen K., Stotter C., Klestil T., Nehrer S. (2022). Artificial Intelligence in Orthopedic Radiography Analysis: A Narrative Review. Diagnostics.

[19] Murphy E.A., Ehrhardt B., Gregson C.L., von Arx O.A., Hartley A., Whitehouse M.R., Thomas M.S., Stenhouse G., Chesser T.J., Budd C.J. (2022). Machine learning outperforms clinical experts in classification of hip fractures. Sci. Rep..

[20] Jang S.J., Kunze K.N., Vigdorchik J.M., Jerabek S.A., Mayman D.J., Sculco P.K. (2022). John Charnley Award: Deep Learning Prediction of Hip Joint Center on Standard Pelvis Radiographs. J. Arthroplast..

[21] Rouzrokh P., Wyles C.C., Philbrick K.A., Ramazanian T., Weston A.D., Cai J.C., Taunton M.J., Lewallen D.G., Berry D.J., Erickson B.J. (2021). A Deep Learning Tool for Automated Radiographic Measurement of Acetabular Component Inclination and Version After Total Hip Arthroplasty. J. Arthroplast..

[22] Yang W., Ye Q., Ming S., Hu X., Jiang Z., Shen Q., He L., Gong X. (2020). Feasibility of automatic measurements of hip joints based on pelvic radiography and a deep learning algorithm. Eur. J. Radiol..

[23] Rouzrokh P., Khosravi B., Johnson Q.J., Faghani S., Vera Garcia D.V., Erickson B.J., Maradit Kremers H., Taunton M.J., Wyles C.C. (2022). Applying Deep Learning to Establish a Total Hip Arthroplasty Radiography Registry: A Stepwise Approach. J. Bone Jt. Surg. Am..

[24] Nagendran M., Chen Y., Lovejoy C.A., Gordon A.C., Komorowski M., Harvey H., Topol E.J., Ioannidis J.P., Collins G.S., Maruthappu M. (2020). Artificial intelligence versus clinicians: Systematic review of design, reporting standards, and claims of deep learning studies. BMJ.

[25] Taylor-Phillips S., Stinton C. (2019). Fatigue in radiology: A fertile area for future research. Br. J. Radiol..

[26] Stec N., Arje D., Moody A.R., Krupinski E.A., Tyrrell P.N. (2018). A Systematic Review of Fatigue in Radiology: Is It a Problem?. Am. J. Roentgenol..

