# Supplementary material for: Deep Learning for Fully Automated Radiographic Measurements of the Pelvis and Hip
Source: Diagnostics (Basel). 2023 Jan 29;13(3):497. doi: 10.3390/diagnostics13030497 (PMC9914204; doi:10.3390/diagnostics13030497)

Technical details

IB Lab HIPPO Calculation Logic

IB Lab HIPPO performs analyses of either single or bilateral hip images by performing the following steps:

1. An input image is provided
2. A metadata quality check is performed
3. IB Lab HIPPO detects the following information on the input image (**Classifier Model**):
    1. presence of implants:
        1. if no implant is present, continue the calculation
        2. if an implant is present, skip the calculation of the respective side
    2. laterality information for further processing:
        1. AP-LEFT
        2. AP-RIGHT
        3. AP-BILATERAL
4. Segmentation masks and landmarks are predicted on single-hip regions of interest (**Segmentation Model**).
5. Landmarks and geometrical measurements (e.g. axes, diameters) derived from the model outputs.
6. Sanity checks are performed, based on the results
7. Based on the landmarks and geometrical measurements, the following hip measurements are computed: CCD, LCE, Tönnis angle, Sharp angle and femoral head extrusion index.
8. If the image was classified as AP_BILATERAL and no implants are present:
    1. The orthograde angle is calculated.
9. If the image is classified as AP_BILATERAL, no implants are present and the teardrop landmarks are detected on both sides: The LCE angle calculation is corrected by the hip axis defined by the connecting line between the teardrop landmarks.
10. All results are collected in a graphical report.

IB Lab HIPPO Network Architecture Description

IB Lab HIPPO is comprised of multiple convolutional deep neural networks (CNNs) which operate on either all or part of the input images and perform segmentation, landmarking and detection tasks. Development environments used include tensorflow 1.15.2, keras 2.2.4 and python 3.6.

The models include:

Classifier Model

- Convolutional deep neural network, containing convolutional layers and batch normalization. The output activation is a softmax function.
- Number of parameters: 8,020,486
- Steps:
    - Load image
    - Image preprocessing, including resizing, spectrum normalization
    - Perform network computations
    - Interpret output
- Training-time image augmentation includes
    - brightness variations
    - contrast variations
    - rotation
    - scaling
    - shifting

Segmentation Model

- Fully convolutional deep neural network of the UNet family.
- Number of parameters: 2,155,031
- Steps:
    - Load image
    - Image preprocessing, including resizing, spectrum normalization
    - Perform network computations
    - Postprocess and correct output
- Training-time image augmentation includes
    - brightness variations
    - contrast variations
    - rotation
    - scaling
    - shifting

Logic Diagram

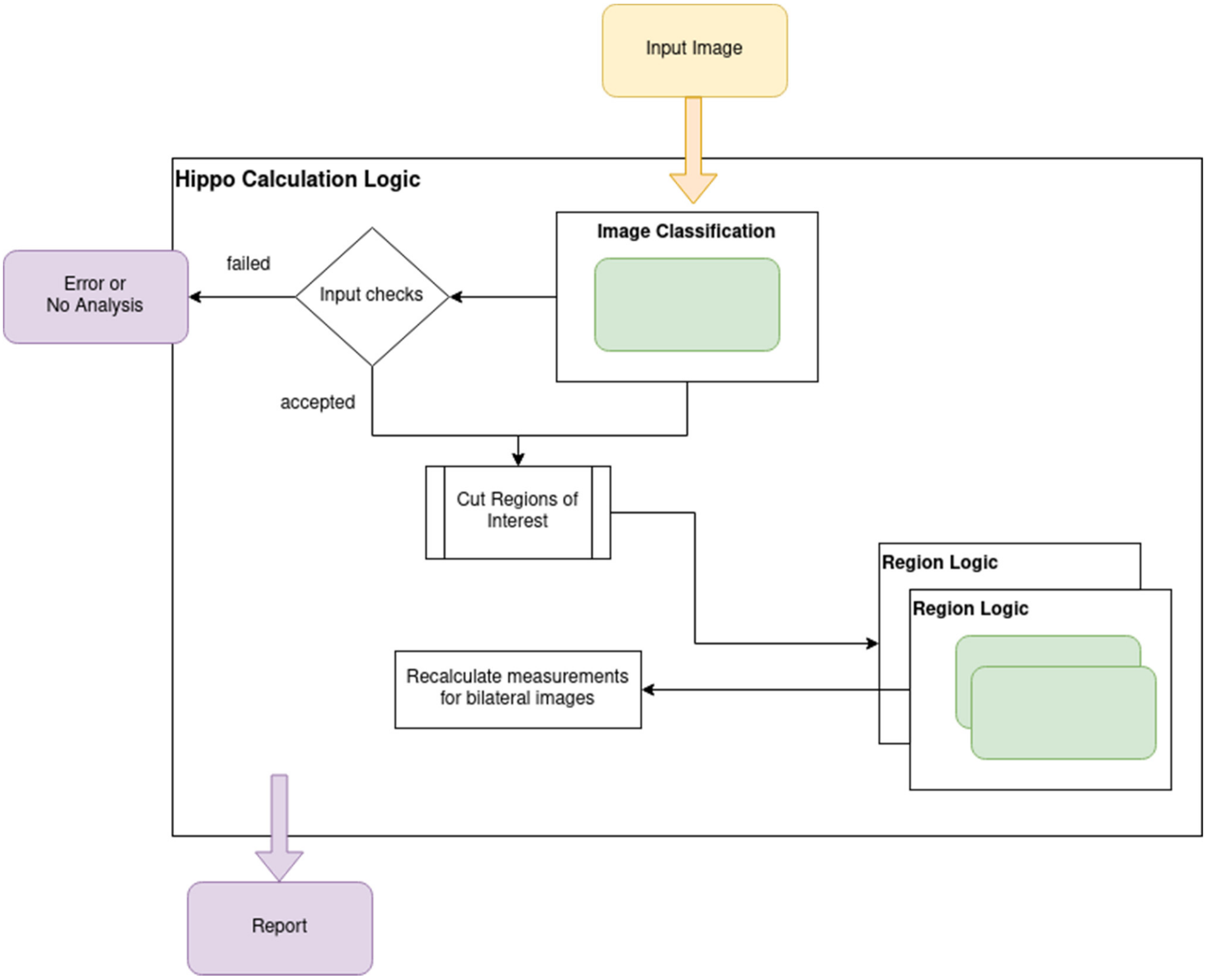

Supplement: Supplementary file 1 [file diagnostics-13-00497-s001.zip › diagnostics-1868868_Supplement File S1.pdf]
