# Supplementary material for: Deep Learning for Fully Automated Radiographic Measurements of the Pelvis and Hip
Source: Diagnostics (Basel). 2023 Jan 29;13(3):497. doi: 10.3390/diagnostics13030497 (PMC9914204; doi:10.3390/diagnostics13030497)
Supplement: Supplementary file 1 [file diagnostics-13-00497-s001.zip › diagnostics-1868868_Supplement File S2.pdf]

Supplement S2

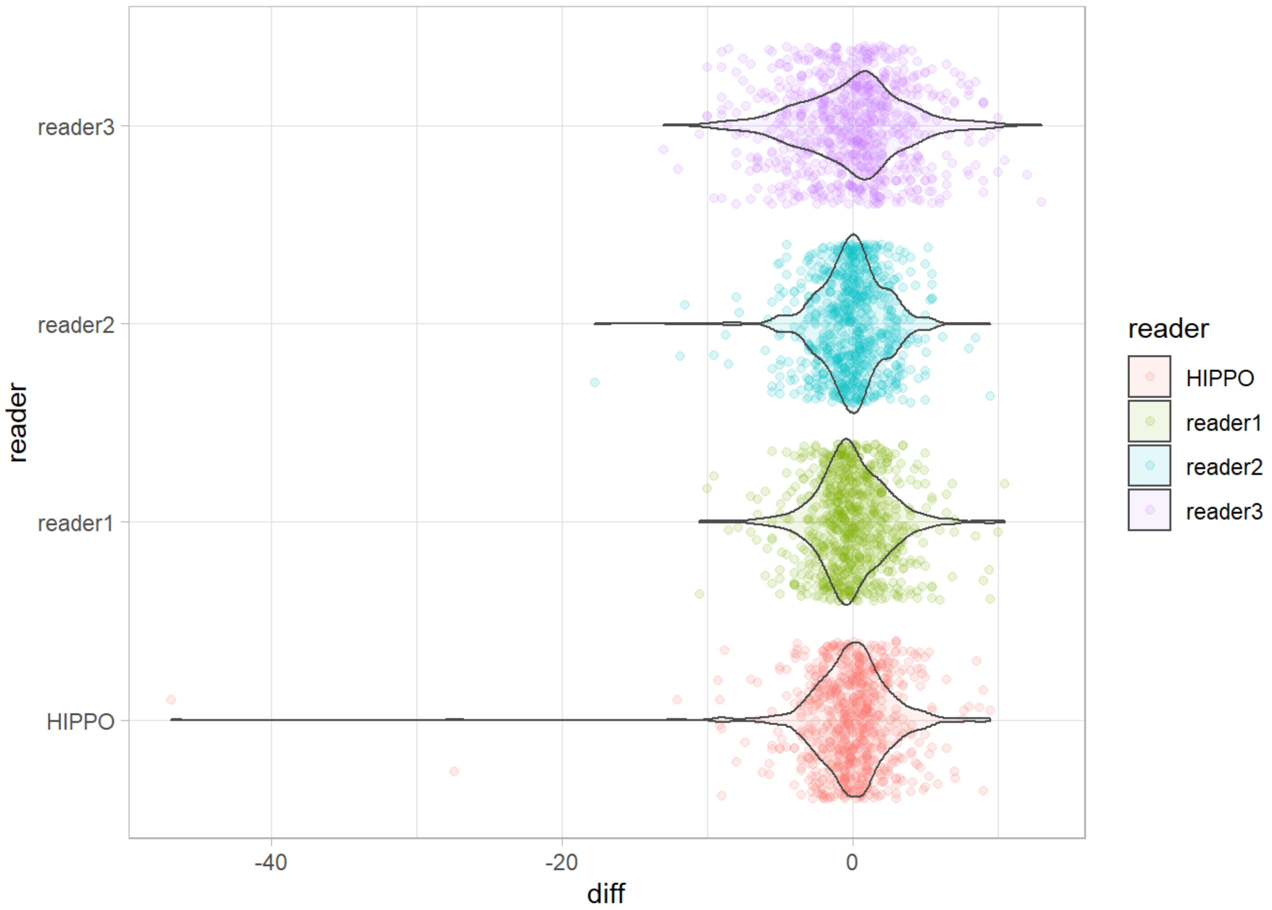

Figure S1: Deviation from the median for each individual observation for all readers and HIPPO™ including outliers

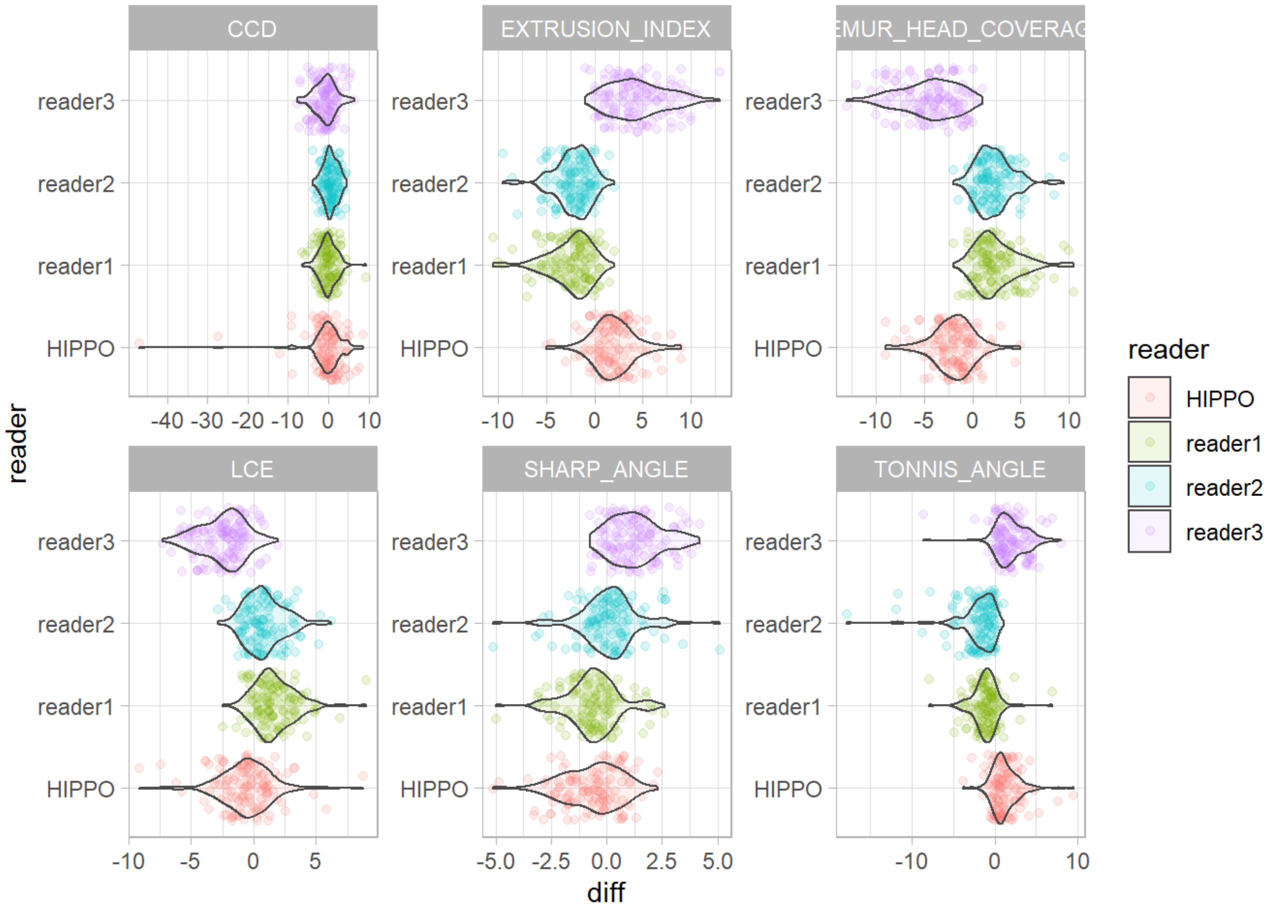

Figure S2: Deviations from the median for each individual observation for all measurements for reader 1-3 and HIPPO™ including outliers

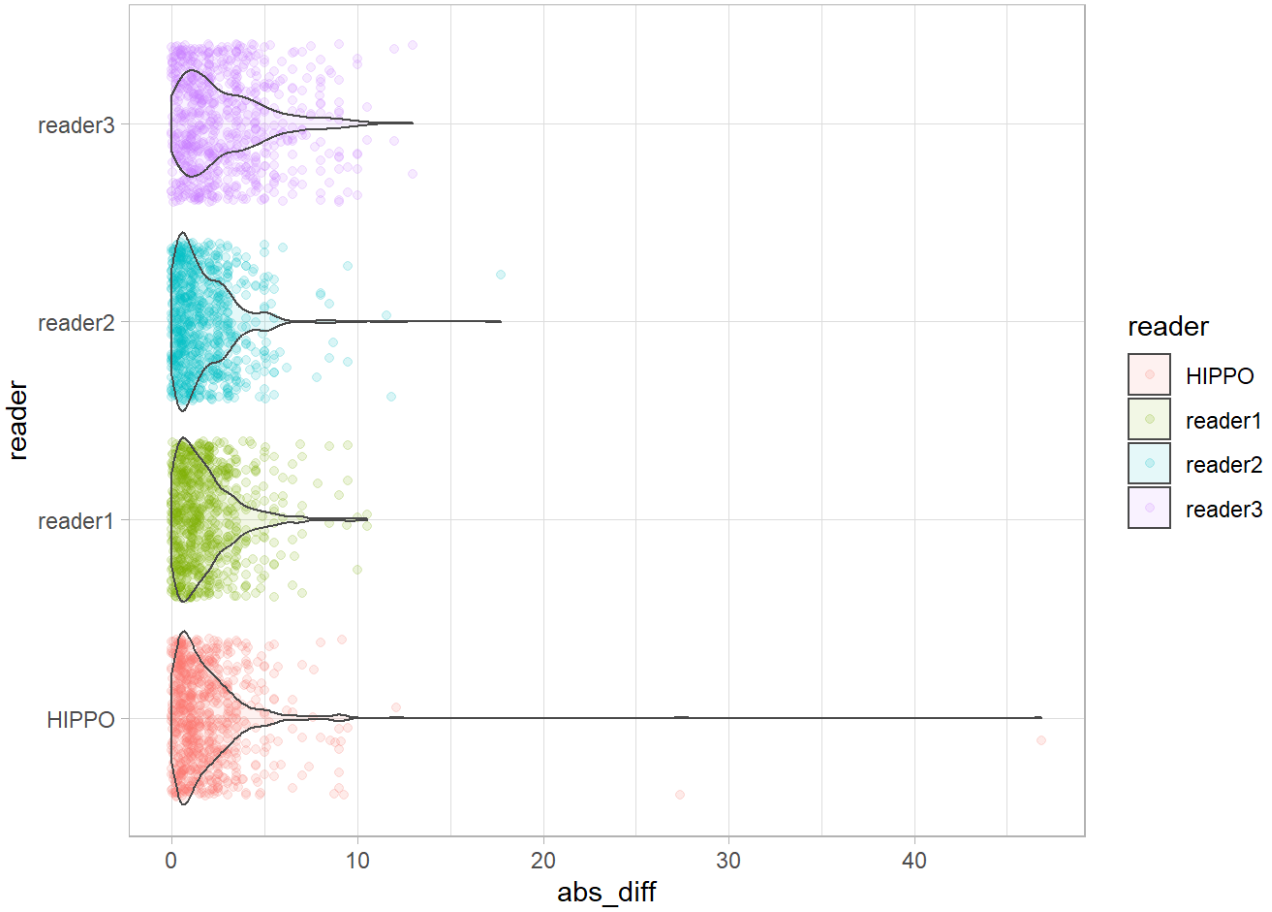

Figure S3: Absolute deviation from the median for each individual observation for all readers and HIPPO™ including outliers

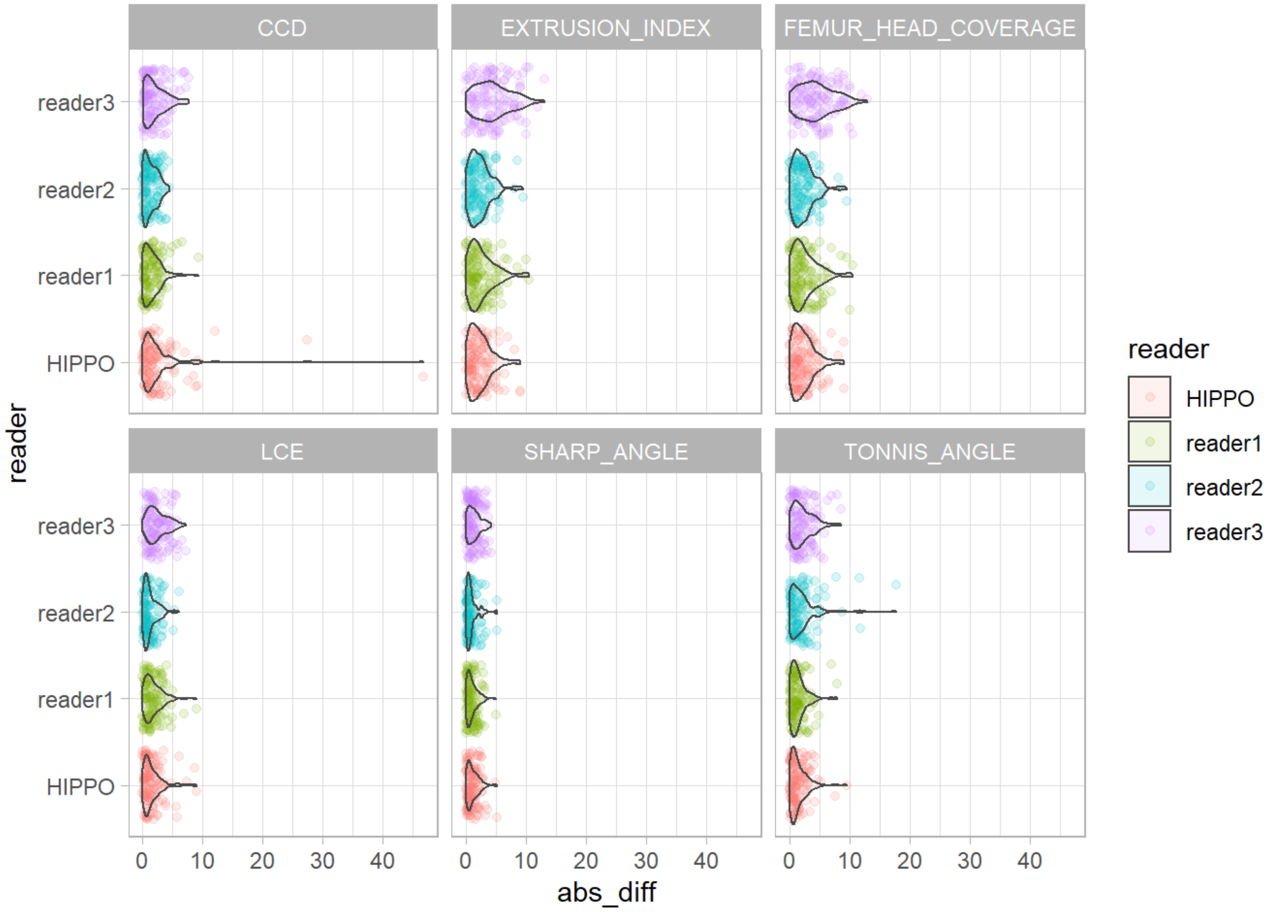

Figure S4: Absolute deviation from the median for all measurements for all readers and HIPPO™ including outliers

CCD

	reader	rank	perc	perc_better
1	HIPPO	1	0	0
2	HIPPO	2	0,9	0,9
3	HIPPO	3	41,575	42,475
4	HIPPO	4	57,525	100
5	reader1	1	18,7	18,7
6	reader1	2	79,05	97,75
7	reader1	3	2,15	99,9
8	reader1	4	0,1	100
9	reader2	1	81,275	81,275
10	reader2	2	18,55	99,825
11	reader2	3	0,175	100
12	reader2	4	0	100
13	reader3	1	0,025	0,025
14	reader3	2	1,5	1,525
15	reader3	3	56,1	57,625
16	reader3	4	42,375	100

EXTRUSION_INDEX

	reader	rank	perc	perc_better
1	HIPPO	1	39,9	39,9
2	HIPPO	2	47,875	87,775
3	HIPPO	3	12,225	100
4	HIPPO	4	0	100
5	reader1	1	4,4	4,4
6	reader1	2	15,95	20,35
7	reader1	3	79,65	100
8	reader1	4	0	100
9	reader2	1	55,7	55,7
10	reader2	2	36,175	91,875
11	reader2	3	8,125	100
12	reader2	4	0	100
13	reader3	1	0	0
14	reader3	2	0	0
15	reader3	3	0	0
16	reader3	4	100	100

FEMUR_HEAD_COVERAGE

	reader	rank	perc	perc_better
1	HIPPO	1	40,675	40,675
2	HIPPO	2	46,45	87,125
3	HIPPO	3	12,875	100
4	HIPPO	4	0	100
5	reader1	1	3,5	3,5
6	reader1	2	16,775	20,275
7	reader1	3	79,725	100
8	reader1	4	0	100
9	reader2	1	55,825	55,825
10	reader2	2	36,775	92,6
11	reader2	3	7,4	100
12	reader2	4	0	100
13	reader3	1	0	0
14	reader3	2	0	0
15	reader3	3	0	0
16	reader3	4	100	100

LCE

	reader	rank	perc	perc_better
1	HIPPO	1	8,025	8,025
2	HIPPO	2	71,425	79,45
3	HIPPO	3	20,55	100
4	HIPPO	4	0	100
5	reader1	1	0,875	0,875
6	reader1	2	19,95	20,825
7	reader1	3	79,1	99,925
8	reader1	4	0,075	100
9	reader2	1	91,1	91,1
10	reader2	2	8,625	99,725
11	reader2	3	0,275	100
12	reader2	4	0	100
13	reader3	1	0	0
14	reader3	2	0	0
15	reader3	3	0,075	0,075
16	reader3	4	99,925	100

SHARP_ANGLE

	reader	rank	perc	perc_better
1	HIPPO	1	10,075	10,075
2	HIPPO	2	24,725	34,8
3	HIPPO	3	52,85	87,65
4	HIPPO	4	12,35	100
5	reader1	1	28,2	28,2
6	reader1	2	45	73,2
7	reader1	3	23,625	96,825
8	reader1	4	3,175	100
9	reader2	1	61,15	61,15
10	reader2	2	27,975	89,125
11	reader2	3	10,025	99,15
12	reader2	4	0,85	100
13	reader3	1	0,575	0,575
14	reader3	2	2,3	2,875
15	reader3	3	13,5	16,375
16	reader3	4	83,625	100

TONNIS_ANGLE

	reader	rank	perc	perc_better
1	HIPPO	1	42,875	42,875
2	HIPPO	2	56,5	99,375
3	HIPPO	3	0,625	100
4	HIPPO	4	0	100
5	reader1	1	57,1	57,1
6	reader1	2	42,775	99,875
7	reader1	3	0,125	100
8	reader1	4	0	100
9	reader2	1	0,025	0,025
10	reader2	2	0,475	0,5
11	reader2	3	67,6	68,1
12	reader2	4	31,9	100
13	reader3	1	0	0
14	reader3	2	0,25	0,25
15	reader3	3	31,65	31,9
16	reader3	4	68,1	100

Figure S5: Data for all measurements and readers for SUCRA plots, "perc_better" indicates the probability for the reader to rank this place or better
